# Injuries by Events in Combined Events (Decathlon and Heptathlon) During 11 International Outdoor Athletics Championships

**DOI:** 10.1111/sms.70142

**Published:** 2025-10-07

**Authors:** Pascal Edouard, Karsten Hollander

**Affiliations:** ^1^ Laboratoire Interuniversitaire de Biologie de la Motricité Université Jean Monnet Saint‐Etienne, Lyon 1, Université Savoie Mont‐Blanc Saint‐Etienne France; ^2^ Department of Clinical and Exercise Physiology, Sports Medicine Unity University Hospital of Saint‐Etienne, Faculty of Medicine Saint‐Etienne France; ^3^ European Athletics Medical & Anti‐Doping Commission European Athletics Association (EAA) Lausanne Switzerland; ^4^ Institute of Interdisciplinary Exercise Science and Sports Medicine MSH Medical School Hamburg Hamburg Germany

**Keywords:** decathlon, epidemiology, heptathlon, performance, sports injury, track and field

## Abstract

We aimed to determine whether some events carry a higher risk of injuries than others in combined events (decathlon and heptathlon) during international outdoor Athletics championships. A secondary aim was also to describe injury characteristics according to the different events. We conducted a total population study. During decathlon and heptathlon of 11 international outdoor Athletics championships from 2007 to 2024, in‐competition injuries were collected by medical teams and local organizing committees. We performed descriptive analyses and calculated injury incidence rates by events. A total of 66 in‐competition injuries and 2901 starts were reported for decathlon, and 67 in‐competition injuries and 2063 starts for heptathlon. The proportion of injuries varied across events, with higher proportions in pole vault (19.7%) and high jump (18.2%) for decathlon, and in long jump (23.9%) and 800 m (16.4%) for heptathlon. The injury incidence rates varied across events, with higher rates in pole vault (46.3 (95% CI: 21.7 to 70.8)) and high jump (38.2 (95% CI: 17.0 to 59.4)) for decathlon, and in long jump (54.1 (95% CI: 28.3 to 79.8)) for heptathlon. The distribution of injury characteristics (i.e., location, type, mode of onset, severity) varied according to the events for decathlon and heptathlon. In conclusion, explosive jumping events had a higher risk of injuries than others within combined: pole vault and high jump for decathlon, and long jump for heptathlon. Injury characteristics varied according to the events. Our present study provides information that could help plan medical services during competitions of combined events and prepare athletes to face the injury challenge of combined events.

## 1 Introduction

In Athletics, combined events, such as decathlon for men and heptathlon for women in outdoor competitions, are a discipline challenging athletes to demonstrate excellence across a diverse range of running, jumping, and throwing events. Furthermore, combined events are the discipline of Athletics with the highest injury incidence rate during international outdoor Athletics championships (235 injuries per 1000 registered athletes for decathlon and 241 for heptathlon) [1]. As in other Athletics disciplines and in other sports, such injuries may negatively affect athletes' health [1] and also performance [2]. To both protect athletes' health and improve athletes' performance, there is an interest in continuing efforts toward injury risk reduction in combined events, focusing on competition settings [2].

A better understanding of the extent of the injury problem represents one option [3]. Previous studies reported data on injuries in combined events; however, over one or more seasons and not within a competition setting [4, 5, 6, 7], and with a retrospective data collection approach [4, 5, 6] or with a prospective approach but without a description of injury characteristics [7]. Only few studies have examined competitions, and these reported injury frequency, rate, and/or characteristics for decathlon and heptathlon as a whole, combining injuries from all 10 or 7 events, respectively [1, 2, 8, 9]. However, given the different physical, mechanical, technical, and psychological demands between these events, as for the different disciplines within Athletics [1], the injury risk and characteristics could substantially differ between events. In addition, Edouard et al. [2] suggested that the higher injury incidence rate reported in combined events could be in part explained by the combination of the injury risks of the events. Thus, to improve injury knowledge and, in turn, injury risk reduction and performance strategies [2], a deeper analysis of injuries occurring during decathlon and heptathlon competitions, broken down by individual event, would provide valuable insights.

In this context, our primary aim was to determine whether some events carry a higher risk of injuries than others in combined events (decathlon and heptathlon) during international outdoor Athletics championships. A secondary aim was also to describe injury characteristics according to the different events.

## 2 Methods

### 2.1 Study Design and Overall Procedure

In this total population study, we analyzed in‐competition injury data prospectively collected in elite athletes participating in combined events in at least one of the 11 following international outdoor Athletics championships: Olympic Games 2012 [10]; World Outdoor Championships 2007 [11], 2009 [12], 2011 [13], and 2013 [14]; and European Outdoor Championships 2012 [15], 2014, 2016, 2018, 2022 [16], and 2024 [17].

Before each championship, athletes were informed about the study and that they could refuse the use of their data [1, 2]. All injuries reported to the database were pseudo‐anonymous. The study protocol was reviewed and approved by the Saint‐Etienne University Hospital Ethics Committee (Institutional Review Board: IORG0007394; IRBN742020/CHUSTE).

### 2.2 Injury and Exposure Definition and Data Collection

We used injury data prospectively collected using the same study design, injury definition, and data collection procedures previously described in detail [1, 2]. Injuries were prospectively collected by the national medical teams (physicians and/or physiotherapists) and/or by the local organizing committee physicians (LOC) daily during each championship [1, 2]. Injuries were defined as “all musculoskeletal injuries (traumatic and overuse) and concussion newly incurred during competition or training, regardless of the consequences with respect to the athlete's absence from competition or training” [1, 2, 18]. If a single incident resulted in more than one injured body part and/or type of injury, each body part and/or type of injury was counted as a separate injury [1, 2]. We included in the present study only injuries occurring during competition (injuries during warm‐up were considered as in‐competition injuries; training injuries were excluded) of decathlon and heptathlon. Injury characteristics were described using previously used classifications for location (i.e., head, trunk, upper extremity, hip & groin, thigh, knee, lower leg, Achilles tendon, ankle, and foot), type (i.e., muscle, tendon, ligament, bone, articular not ligament, skin, and others), mode of onset, and severity [1, 2, 18]. Injury severity was assessed by the estimated number of days of absence from sport that the physician or physiotherapist reported when reporting the injury, without any tracking of the exact date of return to sport [1, 2, 11, 18].

We used as exposure measurement: the *starts* (i.e., sum of athletes starting in each event, including “did not finish” (DNF), “no mark point” (NM), “disqualified” (DQ), and the first “did not start” (DNS)) [2, 11], extracted for each decathlon and heptathlon competition (1 start per athlete competing), as well as for each of their respective events (max. of 10 starts per decathlete competing and 7 starts per heptathlete competing), from the results list of World Athletics (https://worldathletics.org/competition/calendar‐results) or European Athletics (https://www.european‐athletics.com).

### 2.3 Data Analysis

We performed a descriptive analysis and presented the data using frequency with percentage for categorical data and mean with standard deviation for continuous variables. We reported injury surveillance quality analysis (i.e., national medical team participation, athletes' coverage, and response rate) according to Edouard et al. [19]. We calculated *injury incidence rates*, that is, number of injuries per 1000 athlete starts (with 95% confidence intervals, 95% CI), for in‐competition injuries and in‐competition time‐loss injuries, for a decathlon and a heptathlon, as well as for each of the 10 and 7 events of the decathlon and heptathlon, respectively.

## 3 Results

### 3.1 Population

Over the 11 international outdoor Athletics championships from 2007 to 2024, there was a total of 314 starts for a decathlon and 2901 starts in their 10 events, and 307 starts for a heptathlon and 2063 starts in their seven events (Table 1). On average, 91.3% of the national medical teams, covering 84.6% of the registered athletes, participated in the injury surveillance project, and returned 92.4% of the report forms. No athlete refused the use of their data for scientific research.

**TABLE 1:** Number of starts for a decathlon (men) and a heptathlon (women), number of starts for each of the 10 events of a decathlon and each of the seven events of a heptathlon, number of in‐competition injuries and in‐competition time‐loss injuries (with percentage of the total) for decathlon and heptathlon as well as their respective events, and number of in‐competition injuries and in‐competition time‐loss injuries per 1000 starts for decathlon and heptathlon as well as their respective events during the 11 international outdoor Athletics championships.

		Total (decathlon)	First day					Second day				
			100 m	Long jump	Shot put	High jump	400 m	110 m hurdles	Discus throw	Pole vault	Javelin throw	1500 m
Decathlon (men)	Number of starts	314	314	314	314	314	303	281	280	281	256	244
	Number of injuries (*n* (%))	66 (100.0)	6 (9.1)	11 (16.7)	1 (1.5)	12 (18.2)	4 (6.1)	5 (7.6)	2 (3.0)	13 (19.7)	4 (6.1)	8 (12.1)
	Number of injuries per 1000 starts (95% CI)	210.2 (165.1–255.3)	19.1 (4.0–34.3)	35.0 (14.7–55.4)	3.2 (0.0–9.4)	38.2 (17.0–59.4)	13.2 (0.3–26.1)	17.8 (2.3–33.3)	7.1 (0.0–17.0)	46.3 (21.7–70.8)	15.6 (0.4–30.8)	32.8 (10.4–55.1)
	Number of time‐loss injuries (*n* (%))	36 (100.0)	4 (11.1)	4 (11.1)	1 (2.8)	7 (19.4)	3 (8.3)	3 (8.3)	2 (5.6)	7 (19.4)	4 (11.1)	1 (2.8)
	Number of time‐loss injuries per 1000 starts (95% CI)	114.6 (79.4–149.9)	12.7 (0.3–25.1)	12.7 (0.3–25.1)	3.2 (0.0–9.4)	22.3 (6.0–38.6)	9.9 (0.0–21.0)	10.7 (0.0–22.7)	7.1 (0.0–17.0)	24.9 (6.7–43.1)	15.6 (0.4–30.8)	4.1 (0.0–12.1)

		Total (heptathlon)	100 m Hurdles	High Jump	Shot Put	200 m	Long Jump	Javelin Throw	800 m
Hepathlon (women)	Number of starts	307	307	307	306	304	296	288	255
	Number of injuries (*n* (%))	67 (100.0)	10 (14.9)	9 (13.4)	4 (6.0)	7 (10.4)	16 (23.9)	10 (14.9)	11 (16.4)
	Number of injuries per 1000 starts (95% CI)	218.2 (172.0–264.4)	32.6 (12.7–52.4)	29.3 (10.4–48.2)	13.1 (0.3–25.8)	23.0 (6.2–39.9)	54.1 (28.3–79.8)	34.7 (13.6–55.9)	43.1 (18.2–68.1)
	Number of time‐loss injuries (*n* (%))	41 (100.0)	4 (9.8)	6 (14.6)	2 (4.9)	3 (7.3)	14 (34.1)	8 (19.5)	4 (9.8)
	Number of time‐loss injuries per 1000 starts (95% CI)	133.6 (95.5–171.6)	13.0 (0.3–25.7)	19.5 (4.1–35.0)	6.5 (0.0–15.6)	9.9 (0.0–21.0)	47.3 (23.1–71.5)	27.8 (8.8–46.8)	15.7 (0.4–30.9)

### 3.2 In‐Competition Injuries in Combined Events

During the 11 championships, a total of 66 in‐competition injuries were reported in decathlon and 67 in heptathlon. Of them, 36 (54.5%) were in‐competition time‐loss injuries in decathlon and 41 (61.2%) in heptathlon (Table 1). The number and proportion of injuries and time‐loss injuries varied across events for decathlon and heptathlon, with higher proportions in pole vault (19.7% and 19.4%, respectively) and high jump (18.2% and 19.4%, respectively) for decathlon, and in long jump (23.9% and 34.1%, respectively) and 800 m (16.4% and 9.8%, respectively) for heptathlon (Table 1). The order of events classified according to the injury proportion differed between injuries and time‐loss injuries (Table 1).

**FIGURE 1:**
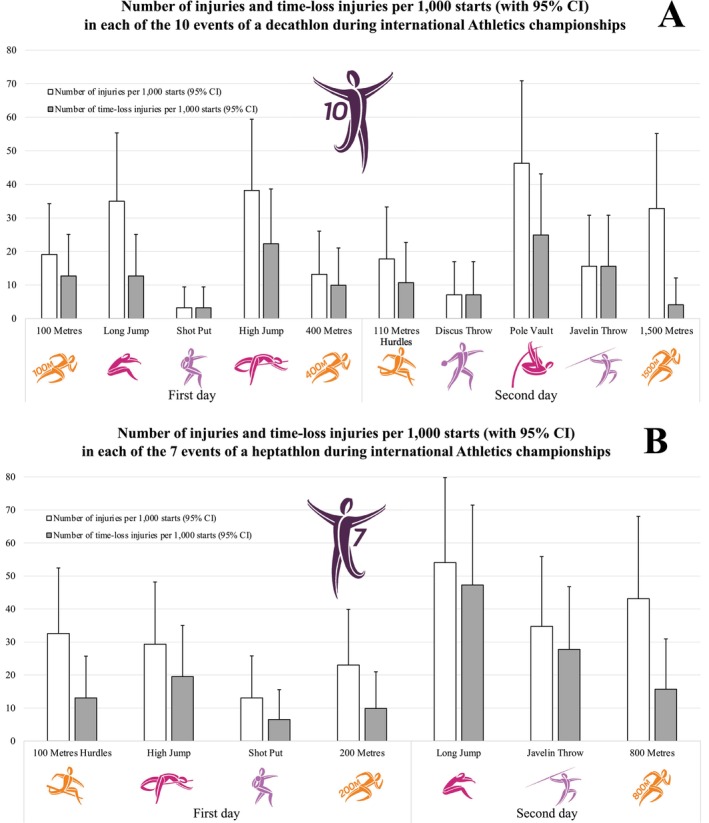
Number of in‐competition injuries and in‐competition time‐loss injuries per 1000 starts for each of the 10 events of a decathlon (A) and each of the seven events of a heptathlon (B), during the 11 international outdoor Athletics championships from 2007 to 2024.

The injury incidence rates were 210.2 (95% CI: 165.1 to 255.3) injuries per 1000 starts in decathlon and 218.2 (95% CI: 172.0 to 264.4) injuries per 1000 starts in heptathlon, and 114.6 (95% CI: 79.4 to 149.9) time‐loss injuries per 1000 starts in decathlon and 133.6 (95% CI: 95.5 to 171.6) time‐loss injuries per 1000 starts in heptathlon (Table 1). The injury incidence rates varied across events, with higher rates in pole vault and high jump for decathlon, and in long jump for heptathlon (Table 1 and Figure 1). The order of events classified according to the injury incidence rate differed between injuries and time‐loss injuries (Table 1).

The distribution of injury characteristics (i.e., location, type, mode of onset, severity) varied according to the events, for decathlon (Table 2) and heptathlon (Table 3). However, no between‐events statistical comparisons were performed on the injury characteristics, given the small numbers of each injury characteristic. Most injuries in decathlon were traumatic injuries (50%), to the muscle (30.3%), skin (22.7%), or tendon (21.2%), occurring at the thigh (22.7%) and the ankle (16.7%). In heptathlon, most injuries were traumatic (46.3%), to the muscle (40.3%), ligaments (26.9%) or tendons (10.4%), occurring at the thigh (20.9%) or the knee (14.9%).

**TABLE 2:** Characteristics of in‐competition injuries (i.e., location, type, mode of onset, severity), presented in number with percentage, for a decathlon as well as its 10 respective events, during the 11 international outdoor Athletics championships.

	Total	First day					Second day				
		100 m	Long jump	Shot put	High jump	400 m	110 m Hurdles	Discus	Pole vault	Javelin	1500 m
Total	66 (100.0)	6 (9.1)	11 (16.7)	1 (1.5)	12 (18.2)	4 (6.1)	5 (7.6)	2 (3.0)	13 (19.7)	4 (6.1)	8 (12.1)
Location											
Head	4 (6.1)	0 (0.0)	0 (0.0)	0 (0.0)	0 (0.0)	0 (0.0)	0 (0.0)	0 (0.0)	3 (23.1)	0 (0.0)	1 (12.5)
Trunk	2 (3.0)	0 (0.0)	0 (0.0)	0 (0.0)	0 (0.0)	1 (25.0)	0 (0.0)	0 (0.0)	1 (7.7)	0 (0.0)	0 (0.0)
Upper extremity	7 (10.6)	0 (0.0)	0 (0.0)	0 (0.0)	1 (8.3)	0 (0.0)	0 (0.0)	0 (0.0)	1 (7.7)	**4 (100.0)**	1 (12.5)
Hip & groin	5 (7.6)	1 (16.7)	1 (9.1)	0 (0.0)	1 (8.3)	0 (0.0)	**2 (40.0)**	0 (0.0)	0 (0.0)	0 (0.0)	0 (0.0)
Thigh	**15 (22.7)**	**3 (50.0)**	2 (18.2)	0 (0.0)	1 (8.3)	**2 (50.0)**	**2 (40.0)**	**1 (50.0)**	**4 (30.8)**	0 (0.0)	0 (0.0)
Knee	7 (10.6)	1 (16.7)	1 (9.1)	0 (0.0)	2 (16.7)	1 (25.0)	0 (0.0)	0 (0.0)	1 (7.7)	0 (0.0)	1 (12.5)
Lower leg	3 (4.5)	0 (0.0)	1 (9.1)	**1 (100.0)**	1 (8.3)	0 (0.0)	0 (0.0)	0 (0.0)	0 (0.0)	0 (0.0)	0 (0.0)
Achilles tendon	5 (7.6)	0 (0.0)	1 (9.1)	0 (0.0)	0 (0.0)	0 (0.0)	0 (0.0)	0 (0.0)	1 (7.7)	0 (0.0)	**3 (37.5)**
Ankle	11 (16.7)	1 (16.7)	**4 (36.4)**	0 (0.0)	**4 (33.3)**	0 (0.0)	0 (0.0)	0 (0.0)	1 (7.7)	0 (0.0)	1 (12.5)
Foot	7 (10.6)	0 (0.0)	1 (9.1)	0 (0.0)	2 (16.7)	0 (0.0)	1 (20.0)	**1 (50.0)**	1 (7.7)	0 (0.0)	1 (12.5)
Type											
Muscle	**20 (30.3)**	**3 (50.0)**	**3 (27.3)**	0 (0.0)	2 (16.7)	**3 (75.0)**	1 (20.0)	**1 (50.0)**	**5 (38.5)**	**2 (50.0)**	0 (0.0)
Tendon	14 (21.2)	2 (33.3)	2 (18.2)	**1 (100.0)**	2 (16.7)	0 (0.0)	**2 (40.0)**	0 (0.0)	0 (0.0)	1 (25.0)	**4 (50.0)**
Ligament	9 (13.6)	1 (16.7)	2 (18.2)	0 (0.0)	2 (16.7)	0 (0.0)	0 (0.0)	0 (0.0)	2 (15.4)	1 (25.0)	1 (12.5)
Bone	1 (1.5)	0 (0.0)	0 (0.0)	0 (0.0)	0 (0.0)	0 (0.0)	0 (0.0)	0 (0.0)	1 (7.7)	0 (0.0)	0 (0.0)
Articular not ligament	5 (7.6)	0 (0.0)	1 (9.1)	0 (0.0)	**3 (25.0)**	1 (25.0)	0 (0.0)	0 (0.0)	0 (0.0)	0 (0.0)	0 (0.0)
Skin	15 (22.7)	0 (0.0)	3 (27.3)	0 (0.0)	**3 (25.0)**	0 (0.0)	**2 (40.0)**	**1 (50.0)**	3 (23.1)	0 (0.0)	3 (37.5)
Others	2 (3.0)	0 (0.0)	0 (0.0)	0 (0.0)	0 (0.0)	0 (0.0)	0 (0.0)	0 (0.0)	2 (15.4)	0 (0.0)	0 (0.0)
Mode of onset											
Traumatic	**33 (50.0)**	2 (33.3)	3 (27.3)	0 (0.0)	**9 (75.0)**	**2 (50.0)**	**3 (60.0)**	**1 (50.0)**	**7 (53.8)**	**3 (75.0)**	3 (37.5)
Overuse	24 (36.4)	**4 (66.7)**	**4 (36.4)**	**1 (100.0)**	3 (25.0)	**2 (50.0)**	0 (0.0)	**1 (50.0)**	4 (30.8)	1 (25.0)	**4 (50.0)**
Other	9 (13.6)	0 (0.0)	**4 (36.4)**	0 (0.0)	0 (0.0)	0 (0.0)	2 (40.0)	0 (0.0)	2 (15.4)	0 (0.0)	1 (12.5)
Severity											
No time‐loss	**26 (39.4)**	**2 (33.3)**	**7 (63.6)**	0 (0.0)	**4 (33.3)**	**1 (25.0)**	**3 (60.0)**	0 (0.0)	3 (23.1)	0 (0.0)	**6 (75.0)**
Less than 1 week	9 (13.6)	0 (0.0)	2 (18.2)	0 (0.0)	3 (25.0)	**1 (25.0)**	0 (0.0)	0 (0.0)	0 (0.0)	**2 (50.0)**	1 (12.5)
1 to 4 weeks	13 (19.7)	1 (16.7)	1 (9.1)	**1 (100.0)**	2 (16.7)	**1 (25.0)**	1 (20.0)	**1 (50.0)**	**4 (30.8)**	1 (25.0)	0 (0.0)
More than 4 weeks	13 (19.7)	**2 (33.3)**	1 (9.1)	0 (0.0)	2 (16.7)	**1 (25.0)**	1 (20.0)	**1 (50.0)**	3 (23.1)	1 (25.0)	1 (12.5)
Missing	5 (7.6)	1 (16.7)	0 (0.0)	0 (0.0)	1 (8.3)	0 (0.0)	0 (0.0)	0 (0.0)	3 (23.1)	0 (0.0)	0 (0.0)

*Note:* The percentage values of the first line (“total”) were calculated based on the total number of injuries (*n* = 66), then all percentage values for each column were calculated based on the value reported in the first line of the respective column (“total”). Highlighted in bold is the most frequent injury by location, type, mode of onset, and severity for a decathlon as well as for their 10 respective events.

**TABLE 3:** Characteristics of in‐competition injuries (i.e., location, type, mode of onset, severity), presented in numbers with percentages, for a heptathlon as well as its seven respective events during the 11 international outdoor Athletics championships.

	Total	First day				Second day		
		100 m Hurdles	High jump	Shot put	200 m	Long jump	Javelin	800 m
Total	67 (100.0)	10 (14.9)	9 (13.4)	4 (6.0)	7 (10.4)	16 (23.9)	10 (14.9)	11 (16.4)
Location								
Head	3 (4.5)	0 (0.0)	1 (11.1)	0 (0.0)	0 (0.0)	0 (0.0)	2 (20.0)	0 (0.0)
Trunk	7 (10.4)	0 (0.0)	1 (11.1)	0 (0.0)	0 (0.0)	**3 (18.8)**	2 (20.0)	1 (9.1)
Upper extremity	8 (11.9)	1 (10.0)	0 (0.0)	0 (0.0)	0 (0.0)	**3 (18.8)**	**3 (30.0)**	1 (9.1)
Hip & groin	3 (4.5)	0 (0.0)	1 (11.1)	1 (25.0)	0 (0.0)	0 (0.0)	0 (0.0)	1 (9.1)
Thigh	**14 (20.9)**	**4 (40.0)**	0 (0.0)	0 (0.0)	**2 (28.6)**	**3 (18.8)**	0 (0.0)	**5 (45.5)**
Knee	10 (14.9)	0 (0.0)	2 (22.2)	**3 (75.0)**	1 (14.3)	**3 (18.8)**	1 (10.0)	0 (0.0)
Lower leg	7 (10.4)	1 (10.0)	1 (11.1)	0 (0.0)	**2 (28.6)**	0 (0.0)	0 (0.0)	3 (27.3)
Achilles tendon	3 (4.5)	1 (10.0)	0 (0.0)	0 (0.0)	0 (0.0)	1 (6.3)	1 (10.0)	0 (0.0)
Ankle	6 (9.0)	1 (10.0)	**3 (33.3)**	0 (0.0)	0 (0.0)	1 (6.3)	1 (10.0)	0 (0.0)
Foot	6 (9.0)	2 (20.0)	0 (0.0)	0 (0.0)	**2 (28.6)**	2 (12.5)	0 (0.0)	0 (0.0)
Type								
Muscle	**27 (40.3)**	**6 (60.0)**	**2 (22.2)**	**2 (50.0)**	**4 (57.1)**	4 (25.0)	0 (0.0)	**9 (81.8)**
Tendon	7 (10.4)	2 (20.0)	1 (11.1)	0 (0.0)	0 (0.0)	3 (18.8)	1 (10.0)	0 (0.0)
Ligament	18 (26.9)	1 (10.0)	**2 (22.2)**	0 (0.0)	1 (14.3)	**7 (43.8)**	**7 (70.0)**	0 (0.0)
Bone	3 (4.5)	0 (0.0)	0 (0.0)	0 (0.0)	1 (14.3)	1 (6.3)	0 (0.0)	1 (9.1)
Articular not ligament	2 (3.0)	0 (0.0)	1 (11.1)	1 (25.0)	0 (0.0)	0 (0.0)	0 (0.0)	0 (0.0)
Skin	5 (7.5)	1 (10.0)	**2 (22.2)**	0 (0.0)	1 (14.3)	1 (6.3)	0 (0.0)	0 (0.0)
Others	5 (7.5)	0 (0.0)	1 (11.1)	1 (25.0)	0 (0.0)	0 (0.0)	2 (20.0)	1 (9.1)
Mode of onset								
Traumatic	**31 (46.3)**	**5 (50.0)**	**6 (66.7)**	1 (25.0)	**4 (57.1)**	**8 (50.0)**	4 (40.0)	3 (27.3)
Overuse	24 (35.8)	3 (30.0)	2 (22.2)	**2 (50.0)**	2 (28.6)	3 (18.8)	**5 (50.0)**	**7 (63.6)**
Other	11 (16.4)	2 (20.0)	1 (11.1)	1 (25.0)	0 (0.0)	5 (31.3)	1 (10.0)	1 (9.1)
Missing	1 (1.5)	0 (0.0)	0 (0.0)	0 (0.0)	1 (14.3)	0 (0.0)	0 (0.0)	0 (0.0)
Severity								
No time‐loss	**23 (34.3)**	**5 (50.0)**	2 (22.2)	**2 (50.0)**	**3 (42.9)**	2 (12.5)	2 (20.0)	**7 (63.6)**
Less than 1 week	14 (20.9)	0 (0.0)	0 (0.0)	1 (25.0)	1 (14.3)	3 (18.8)	**5 (50.0)**	4 (36.4)
1–4 weeks	16 (23.9)	3 (30.0)	**4 (44.4)**	1 (25.0)	0 (0.0)	**5 (31.3)**	3 (30.0)	0 (0.0)
More than 4 weeks	11 (16.4)	1 (10.0)	2 (22.2)	0 (0.0)	2 (28.6)	6 (37.5)	0 (0.0)	0 (0.0)
Missing	3 (4.5)	1 (10.0)	1 (11.1)	0 (0.0)	1 (14.3)	0 (0.0)	0 (0.0)	0 (0.0)

*Note:* The percentage values of the first line (“total”) were calculated based on the total number of injuries (*n* = 67), then all percentage values for each column were calculated based on the value reported in the first line of the respective column (“total”). Highlighted in bold is the most frequent injury by location, type, mode of onset, and severity for a heptathlon as well as for its seven respective events.

## 4 Discussion

The main findings of the present study were that (1) some events had a higher risk of injuries than others within combined events during international outdoor Athletics championships: pole vault and high jump for decathlon, and long jump for heptathlon, were the events with the highest injury proportion and injury incidence rates, and (2) injury characteristics (i.e., location, type, mode of onset, severity) varied according to the events for decathlon and heptathlon.

### 4.1 Injury Risk During Competition of Combined Events

Our present results showed higher in‐competition injury numbers, proportions, and incidence rates for some events, and especially for the jumping events of decathlon and heptathlon (Table 1).

It seems that some events had higher injury rates and higher time‐loss injury rates (e.g., pole vault, high jump, and long jump for decathlon, and long jump for heptathlon), meaning that during these events, injuries were both frequent and led to potential negative consequences on participation. Such events could represent priority targets for athletes, coaches, and health professionals in a performance‐prevention win‐win approach. The higher injury risk in these events could be considered surprising since, during the same context of international Athletics championships, jumps disciplines were not a discipline with a higher injury risk than sprints and hurdles [1]. However, no detailed description was reported for pole vault, high jump, and long jump separately [1]. Outside of the competition setting, during a one‐season follow‐up, jumps disciplines were also not reported as the discipline with higher injury risk than other disciplines [7, 20]. Some studies reported injury data specifically for pole vault [21, 22, 23, 24] or jumps [25, 26], but without any comparisons of injury rates and/or characteristics with other disciplines. Hypotheses of explanation for higher injury risk in jumping events during competitions of combined events could be that these are technical events requiring an optimal mastery of the technique/gesture/movement to both perform and reduce injury risk [23]. Indeed, biomechanical patterns were reported to be associated with higher injuries in pole vault [24]. In addition, the take‐off and landing phases were reported to be specific movements with high load on the musculoskeletal system and thus more risk of injuries [21, 23, 25, 26]. Enoki et al. [25] also reported higher injury risk in pole vault among jumps and hypothesized that the risk of fall from a great height and the higher run‐up speeds could explain this risk.

Some events had higher injury rates but relatively lower time‐loss injury rates (e.g., 1500 m for decathlon, and 100 m hurdles and 800 m for heptathlon). This could mean that although injuries were reported for these events, the potential consequences on participation were less important. This can be explained because this was the first event of the competition (i.e., 100 m hurdles for heptathlon) and athletes preferred to continue and could try to minimize injury consequences. For 1500 m for decathlon and 800 m for heptathlon, the higher injury rate could be explained by either the cumulative fatigue over the 2 days or over‐reporting of injuries because this is the last event. The lower consequences on participation could be explained by the fact that such events lead to less biomechanical constraints on the musculoskeletal system than explosive events; these are the last events and the injury consequence could have been underestimated, or athletes simply start these last events to be registered on the official mark and be officially ranked.

Some events had lower injury rates but higher time‐loss injury rates (e.g., javelin throw for decathlon, and javelin throw and high jump for heptathlon). This could mean that injuries were less frequent, but when they occurred, they were more severe, leading to potential negative consequences on participation. Thus, such disciplines should also be considered by athletes, coaches, and health professionals in a performance–prevention win‐win approach.

All these data are of interest to help plan medical services during competitions of combined events, and also for athletes and coaches to improve the short‐ and long‐term preparation (e.g., training, conditioning, warm‐up) and increase awareness of the body for such events.

### 4.2 Injury Risk and Performance During Competitions of Combined Events

Interestingly, the events with higher values of numbers, proportions, and incidence rates, along with higher consequences on participation, match with those with a higher proportion of “did not score” (i.e., pole vault, long jump, and hurdles for decathlon, and long jump for heptathlon) [27]. The performance in combined events corresponds to the sum of points earned by the athlete in each event based on their performance [2, 27, 28]. Not scoring in one or more events has a major impact on the overall combined events' performance and athletes' ranking [27, 28]. Therefore, from a performance perspective, it is of great interest to better understand events with a higher risk of “not scoring” and their reasons. Indirectly, our present study provides a hypothesis of explanation for “not scoring” reasons in combined events. As reported in a pilot study on three decathlons [29], injuries could be a reason for “not scoring” in combined events. Although this hypothesis should be confirmed and better explored in future studies, this information could help athletes and coaches in the preparation for the competition of combined events. Specifically, more attention should be paid to the combined events preparation for pole vault and high jump in decathlon, and long jump in heptathlon. For example, during training, this could involve targeted physical conditioning to address the specific physical and biomechanical demands of these events, alongside technical skill development to improve event proficiency and reduce weaknesses or errors that may lead to injury. During competition, this could involve maintaining focus on technical execution and movement quality, monitoring for pain, weakness, or fatigue that could impair motor or biomechanical performance and increase injury risk, and remaining attentive to environmental conditions (e.g., wind, rain).

### 4.3 Injury Characteristics During Competitions of Combined Events

The distribution of injury characteristics varied according to the events for decathlon and heptathlon (Tables 2 and 3). These event‐specific injury characteristics had the same trends as the respective discipline‐specific injury patterns reported in Athletics disciplines outside combined events (i.e., sprints, hurdles, jumps, throws, and middle distances) during international Athletics championships [1]. As Edouard et al. [1] suggested, it seems that specific disciplines (and events) lead to specific constraints, and in turn specific injuries, whatever the circumstances (entire season or championships, or individual disciplines or individual events of combined events). However, caution should be taken in our present results and such interpretation, given the small number of injuries per event.

### 4.4 Methodological Considerations

In addition to limitations previously discussed regarding injury data collection during international Athletics championships [1, 2, 30], we have to acknowledge additional limitations. The small number of injuries, in total and per event, limited statistical comparison, reduced the statistical power, and limited the strength of the between‐events comparisons, especially of the injury characteristics, although this was a total population study over 11 international Athletics championships, including elite athletes. The findings on event‐specific risk should be interpreted as indicative trends rather than definitive differences, given the small number of injuries, and especially for injury characteristics, given the lack of formal statistical comparisons. The injury severity should be interpreted with caution, as it was an estimation without formal tracking of the exact date of return to sport [1, 2, 11], and since it has been reported to have a low inter‐examiner reliability [31]. The exposure was the athlete's participation (i.e., starts) and not a duration, intensity, or type of effort for each event. The limitations inherent to the study design (i.e., retrospective analysis) did not allow for analysis of causality. Finally, we have to acknowledge the risk of unmeasured confounding included in the analyses.

## 5 Perspectives

Our present findings could help athletes and their entourage better manage competitions of combined events and face the challenge of injuries.

As stated by Drew et al. [32] “performance cannot be researched without consideration of the health status of the athlete both during competition and the period prior”. Therefore, injury prevention should be included as part of the performance preparation in a performance‐prevention win‐win strategy [2]. A strategy to compete, complete, and perform at best in combined events could be to adequately prepare i) the events with a higher risk of injuries (e.g., pole vault and high jump in decathlon, and long jump in heptathlon) and/or ii) specific injuries (e.g., injuries of the ankle, thigh, and knee, and involving muscle, ligament, and skin). Currently, and to our knowledge, there are no injury risk reduction strategies scientifically validated in combined events. In Athletics, education [33] and regular neuromuscular exercises [34] have been reported to reduce injury risk. Without scientific evidence, injury risk reduction should follow a multifactorial and multidimensional approach, considering biological/physical, psychological, and societal/environmental aspects [35, 36, 37, 38]. Future studies should explore the efficacy of such an approach to reduce injury risk in combined events, and specifically in competition settings. In addition, a better understanding of the physical, mechanical, technical, and psychological demands of combined events could be of help to better target injury risk reduction strategies. This can be done, for instance, by exploring competition‐related internal and external load, cumulative fatigue, and stress, including sex differences.

Our present results can also be of interest to help plan medical services during competitions in combined events [1].

Finally, our present study also highlighted the need to continue injury data collection in combined events to improve the understanding of the extent of the injury problem. This should be done during competition settings as well as during one or more seasons, and by also exploring physical and psychological long‐term health consequences (e.g., pain, osteoarthritis, mental health, burnout). Given the small number of combined events athletes, multicentric studies and/or international registries could be relevant approaches.

## Author Contributions

P.E. and K.H. conceived the study; P.E. participated in injury data collection; P.E. performed data analyses; P.E. drafted the manuscript; and all co‐authors discussed the analysis, contributed substantially to interpreting the results, provided important revisions, and approved the manuscript. All authors understand that they are accountable for all aspects of the work and ensure the accuracy and integrity of this manuscript. P.E. is the guarantor of the manuscript.

## Disclosure

All athletes registered in combined events at the international Athletics championships were included in this study without any restriction based on sex, race/ethnicity/culture, socioeconomic level, or representation from marginalized groups. The research team included two male senior sports medicine physicians and researchers from two countries in Europe (France and Germany).

## Ethics Statement

The study was reviewed and approved by the Saint‐Etienne University Hospital Ethical Committee (Institutional Review Board: IORG0007394, IRBN742020/CHUSTE).

## Conflicts of Interest

None declared. P.E. is an Associate Editor for the British Journal of Sports Medicine. P.E. and K.H. are Associate Editors for the BMJ Open Sports and Exercise Medicine. P.E. is an Associate Editor for the Scandinavian Journal of Medicine & Science in Sports. K.H. is an Editor for the German Journal of Sports Medicine. K.H. is the Editor for the German Journal of Sports Medicine and Chief Medical Officer of the German Athletics Federation.

## Data Availability

The data that support the findings of this study are available from the corresponding author upon reasonable request.

## References

[1] P. Edouard , L. Navarro , P. Branco , V. Gremeaux , T. Timpka , and A. Junge , “Injury Frequency and Characteristics (Location, Type, Cause and Severity) Differed Significantly Among Athletics ('track and Field’) Disciplines During 14 International Championships (2007‐2018): Implications for Medical Service Planning,” British Journal of Sports Medicine 54 (2020): 159–167, 10.1136/bjsports-2019-100717. 31722935

[2] P. Edouard , L. Navarro , J. Pruvost , P. Branco , and A. Junge , “In‐Competition Injuries and Performance Success in Combined Events During Major International Athletics Championships,” Journal of Science and Medicine in Sport 24 (2021): 152–158, 10.1016/j.jsams.2020.07.011. 32868201

[3] W. van Mechelen , H. Hlobil , and H. C. G. Kemper , “Incidence, Severity, Aetiology and Prevention of Sports Injuries,” Sports Medicine 14 (1992): 82–99. 1509229 10.2165/00007256-199214020-00002

[4] B. Mayr , O. Paar , P. Bernett , and M. Folk , “Sports Injuries and Sports Damage in Decathlon Competitors,” Schweizerische Zeitschrift für Sportmedizin 36 (1988): 39–45. 3368782

[5] S. Wentz , M. Engelhardt , and S. Wentz , “Verletzungsanalyse Und Leistungsparameter Bei Jugendzehnakaempfern,” Deutsche Zeitschrift Für Sportmedizin 48 (1997): 389–394.

[6] P. Edouard , A. Kerspern , J. Pruvost , and J. B. Morin , “Four‐Year Injury Survey in Heptathlon and Decathlon Athletes,” Science & Sports 27 (2012): 345–350, 10.1016/j.scispo.2012.04.002.

[7] J. Jacobsson , T. Timpka , J. Kowalski , et al., “Injury Patterns in Swedish Elite Athletics: Annual Incidence, Injury Types and Risk Factors,” British Journal of Sports Medicine 47 (2013): 941–952, 10.1136/bjsports-2012-091651. 23543425

[8] P. Edouard , P. Samozino , G. Escudier , A. Baldini , and J. B. Morin , “Injuries in Youth and National Combined Events Championships,” International Journal of Sports Medicine 33 (2012): 824–828, 10.1055/s-0031-1301332. 22562740

[9] Y. Hiroshige , H. Watanabe , S. Tomiyama , and H. Kato , “Epidemiological Study of Track‐and‐Field Meets On‐Field Medical Care,” Journal of Sport Rehabilitation 34 (2025): 102–108, 10.1123/jsr.2023-0316. 39379015

[10] L. Engebretsen , T. Soligard , K. Steffen , et al., “Sports Injuries and Illnesses During the London Summer Olympic Games 2012,” British Journal of Sports Medicine 47 (2013): 407–414, 10.1136/bjsports-2013-092380. 23515712

[11] J. M. Alonso , A. Junge , P. Renström , L. Engebretsen , M. Mountjoy , and J. Dvorak , “Sports Injuries Surveillance During the 2007 IAAF World Athletics Championships,” Clinical Journal of Sport Medicine 19 (2009): 26–32, 10.1097/JSM.0b013e318191c8e7. 19124980

[12] J. M. Alonso , P. M. Tscholl , L. Engebretsen , M. Mountjoy , J. Dvorak , and A. Junge , “Occurrence of Injuries and Illnesses During the 2009 IAAF World Athletics Championships,” British Journal of Sports Medicine 44 (2010): 1100–1105, 10.1136/bjsm.2010.078030. 21106775

[13] J. M. Alonso , P. Edouard , G. Fischetto , B. Adams , F. Depiesse , and M. Mountjoy , “Determination of Future Prevention Strategies in Elite Track and Field: Analysis of Daegu 2011 IAAF Championships Injuries and Illnesses Surveillance,” British Journal of Sports Medicine 46 (2012): 505–514, 10.1136/bjsports-2012-091008. 22522588 PMC3371221

[14] J.‐M. Alonso , J. Jacobsson , T. Timpka , et al., “Preparticipation Injury Complaint Is a Risk Factor for Injury: A Prospective Study of the Moscow 2013 IAAF Championships,” British Journal of Sports Medicine 49 (2015): 1118–1124, 10.1136/bjsports-2014-094359. 25716152

[15] P. Edouard , F. Depiesse , P. Branco , and J. M. Alonso , “Analyses of Helsinki 2012 European Athletics Championships Injury and Illness Surveillance to Discuss Elite Athletes Risk Factors,” Clinical Journal of Sport Medicine 24 (2014): 409–415, 10.1097/JSM.0000000000000052. 24326930

[16] P. Edouard , P.‐E. Dandrieux , K. Hollander , and M. Zyskowski , “Injuries and Illnesses at the Munich 2022 European Championships: A Prospective Study of 5,419 Athletes From 52 Countries Involved in 9 Sports,” BMJ Open Sport & Exercise Medicine 10 (2024): e001737, 10.1136/bmjsem-2023-001737. PMC10875512 38374943

[17] P. Edouard , S. Iatropoulos , L. Navarro , P. Branco , K. Hollander , and P. E. Dandrieux , “Educating and Promoting Athletes' Health Protection Through Infographics on Injury and Illness Prevention During an International Competition: Prospective Study During the 2024 European Athletics Championships,” BMJ Open Sport & Exercise Medicine 10 (2024): e002162. 10.1136/bmjsem-2024-002162 PMC11429358 39345831

[18] T. Timpka , J.‐M. Alonso , J. Jacobsson , et al., “Injury and Illness Definitions and Data Collection Procedures for Use in Epidemiological Studies in Athletics (Track and Field): Consensus Statement,” British Journal of Sports Medicine 48 (2014): 483–490, 10.1136/bjsports-2013-093241. 24620036

[19] P. Edouard , P. Branco , J. M. Alonso , and A. Junge , “Methodological Quality of the Injury Surveillance System Used in International Athletics Championships,” Journal of Science and Medicine in Sport 19 (2016): 984–989, 10.1016/j.jsams.2016.03.012. 27210109

[20] D. D'Souza , “Track and Field Athletics Injuries a One‐Year Survey*,” British Journal of Sports Medicine 28 (1994): 197–202. 8000821 10.1136/bjsm.28.3.197 PMC1332067

[21] G. S. Rebella , J. O. Edwards , J. J. Greene , M. T. Husen , and D. C. Brousseau , “A Prospective Study of Injury Patterns in High School Pole Vaulters,” American Journal of Sports Medicine 36 (2008): 913–920, 10.1177/0363546507313571. 18326831

[22] B. P. Boden , M. G. Boden , R. G. Peter , F. O. Mueller , and J. E. Johnson , “Catastrophic Injuries in Pole Vaulters: A Prospective 9‐Year Follow‐Up Study,” American Journal of Sports Medicine 40 (2012): 1488–1494, 10.1177/0363546512446682. 22582223

[23] G. Rebella , “A Prospective Study of Injury Patterns in Collegiate Pole Vaulters,” American Journal of Sports Medicine 43 (2015): 808–815, 10.1177/0363546514564542. 25596615

[24] P. Edouard , H. Sanchez , C. Bourrilhon , S. Homo , J. Frère , and J. Cassirame , “Biomechanical Pole Vault Patterns Were Associated With a Higher Proportion of Injuries,” Frontiers in Sports and Active Living 1 (2019): 20, 10.3389/fspor.2019.00020. 33344944 PMC7739738

[25] S. Enoki , M. Nagao , S. Ishimatsu , T. Shimizu , and R. Kuramochi , “Injuries in Collegiate Track and Field Jumping: A 2‐Year Prospective Surveillance Study,” Orthopaedic Journal of Sports Medicine 9 (2021): 2325967120973397, 10.1177/2325967120973397. 33553444 PMC7841680

[26] H. C. Rhim , R. Reichenbach , T. Afifi , et al., “Epidemiology of Injuries in United States High School Track and Field Jumping Events From 2008–2019,” Physician and Sportsmedicine 53 (2025): 47–54, 10.1080/00913847.2024.2394850. 39158839

[27] P. Edouard , J.‐B. Morin , F. Celli , et al., “Dropout in International Combined Events Competitions,” New Studies in Athletics 24 (2009): 63–68.

[28] F. Zarnowski , “The Nature of Decathlon,” in A Basic Guide to Decathlon, ed. G. M. Horn and C. Gardner (Griffin Publishing Group, 2001), 27–37.

[29] P. Edouard , J. Pruvost , J. L. Edouard , and J. B. Morin , “Causes of Dropouts in Decathlon. A Pilot Study,” Physical Therapy in Sport 11 (2010): 133–135, 10.1016/j.ptsp.2010.07.004. 21055707

[30] P. Edouard , P.‐E. Dandrieux , M. Klöwer , et al., “Association Between Feel‐Like Temperatures and Injury Risk During International Outdoor Athletic Championships: A Prospective Cohort Study on 29 579 Athlete Starts During 10 Championships,” British Journal of Sports Medicine 59 (2025): 36–47, 10.1136/bjsports-2023-108050. 39438035

[31] P. Edouard , A. Junge , M. Kiss‐Polauf , et al., “Interrater Reliability of the Injury Reporting of the Injury Surveillance System Used in International Athletics Championships,” Journal of Science and Medicine in Sport 21 (2018): 894–898, 10.1016/j.jsams.2018.02.001. 29503161

[32] M. K. Drew , B. P. Raysmith , and P. C. Charlton , “Injuries Impair the Chance of Successful Performance by Sportspeople: A Systematic Review,” British Journal of Sports Medicine 51 (2017): 1209–1214, 10.1136/bjsports-2016-096731. 28446456

[33] J. Jacobsson , J. Kowalski , T. Timpka , et al., “Universal Prevention Through a Digital Health Platform Reduces Injury Incidence in Youth Athletics (Track and Field): A Cluster Randomised Controlled Trial,” British Journal of Sports Medicine 57 (2023): 364–370, 10.1136/bjsports-2021-105332. 36564148 PMC9985750

[34] S. Iatropoulos , P. E. Dandrieux , L. Navarro , P.‐. E. Dandrieux , D. Blanco , and P. Edouard , “The Dose–Response Relationship of an Exercise‐Based Injury Prevention Program: A Secondary Analysis of a Randomized Controlled Trial on Athletics (Track‐and‐Field) Athletes Over a 39‐Week Follow‐Up,” Scandinavian Journal of Medicine & Science in Sports 34 (2024): e14720, 10.1111/sms.14720. 39232249

[35] P. Edouard , B. Caumeil , E. Verhagen , G. Guilhem , and A. Ruffault , “Maximising Individualisation of Sports Injury Risk Reduction Approach to Reach Success,” Brazilian Journal of Physical Therapy 26 (2022): 100394. 35526371 10.1016/j.bjpt.2022.100394 PMC9092190

[36] S. Iatropoulos , P. E. Dandrieux , D. Blanco , et al., “Effect of an Unsupervised Multidomain Intervention Integrating Education, Exercises, Psychological Techniques and Machine Learning Feedback, on Injury Risk Reduction in Athletics (Track and Field): Protocol of a Randomised Controlled Trial (I‐ReductAI),” BMJ Open Sport & Exercise Medicine 11 (2025): e002501, 10.1136/bmjsem-2025-002501. PMC11843022 39990118

[37] P. Edouard , P.‐E. Dandrieux , S. Iatropoulos , et al., “Injuries in Athletics (Track and Field): A Narrative Review Presenting the Current Problem of Injuries,” Deutsche Zeitschrift Für Sportmedizin 75 (2024): 132–141, 10.5960/dzsm.2024.601.

[38] P. Edouard , A. Richardson , A. Murray , et al., “Ten Tips to Hurdle the Injuries and Illnesses During Major Athletics Championships: Practical Recommendations and Resources,” Frontiers in Sports and Active Living 1 (2019): 12. 33344936 10.3389/fspor.2019.00012 PMC7739783

